# Vaccine anxieties, vaccine preparedness: Perspectives from Africa in a Covid-19 era

**DOI:** 10.1016/j.socscimed.2022.114826

**Published:** 2022-04

**Authors:** Melissa Leach, Hayley MacGregor, Grace Akello, Lawrence Babawo, Moses Baluku, Alice Desclaux, Catherine Grant, Foday Kamara, Marion Nyakoi, Melissa Parker, Paul Richards, Esther Mokuwa, Bob Okello, Kelley Sams, Khoudia Sow

**Affiliations:** a Institute of Development Studies (IDS), University of Sussex, Brighton, BN1 9RE, UK; b Gulu University, Pece-Laroo Division, Gulu, Uganda; c Njala University, Njala Campus, Bo, Sierra Leone; d London School of Hygiene and Tropical Medicine (LSHTM), Keppel St, London, WC1E 7HT, UK; e Institut de Recherche pour le Développement (IRD), France and Dakar, Senegal; f Centre Régional de Recherche et de Formation à la prise en charge de Fann (CRCF), Dakar, Senegal

**Keywords:** Covid-19, Vaccination, Hesitancy, Anxiety, Preparedness, Africa

## Abstract

Global debates about vaccines as a key element of pandemic response and future preparedness in the era of Covid-19 currently focus on questions of supply, with attention to global injustice in vaccine distribution and African countries as rightful beneficiaries of international de-regulation and financing initiatives such as COVAX. At the same time, vaccine demand and uptake are seen to be threatened by hesitancy, often attributed to an increasingly globalised anti-vaxx movement and its propagation of misinformation and conspiracy, now reaching African populations through a social media ‘infodemic’. Underplayed in these debates are the socio-political contexts through which vaccine technologies enter and are interpreted within African settings, and the crucial intersections between supply and demand. We explore these through a ‘vaccine anxieties’ framework attending to both desires for and worries about vaccines, as shaped by bodily, societal and wider political understandings and experiences. This provides an analytical lens to organise and interpret ethnographic and narrative accounts in local and national settings in Uganda and Sierra Leone, and their (dis)connections with global debates and geopolitics. In considering the socially-embedded reasons why people want or do not want Covid-19 vaccines, and how this intersects with the dynamics of vaccine supply, access and distribution in rapidly-unfolding epidemic situations, we bring new, expanded insights into debates about vaccine confidence and vaccine preparedness.

## Highlights

• Discourses of vaccine hesitancy misrepresent African publics as ignorant or confused.
• Covid-19 vaccine anxieties make sense given disease, social and political experiences.
• Vaccine supply inequities interplay with uptake in dynamics across scales.
• Growing familiarity with Covid-19 vaccines has eroded worries, but not linearly.
• Vaccine preparedness must address health system structures and politics.

## 1 Introduction

Vaccination has come to the forefront of global debate and policy in the second year of the Covid-19 pandemic. The remarkable speed and success of vaccine development has heightened emphasis on ‘vaccinating the world’ as the way to attenuate the current pandemic, with the World Health Organisation (WHO)'s Vaccination Strategy seeking to inoculate 70% of people in all countries by mid-2022 (WHO, 2021). Important supply-focused debates focus on vaccine inequities as a reflection of longstanding, broader inequities in global health, and on initiatives to address them. These commonly position African countries as victims of injustice, with vaccine shortages and distribution the main problem to be overcome. New concepts of ‘vaccine preparedness’ or readiness have thus joined the global health policy lexicon with accompanying tools focused on making sure supplies are ‘rolled out’ effectively once they reach countries.

A parallel, though less prominent, debate addresses the concern that populations offered vaccines might not actually take them up. The key concept here is vaccine hesitancy, defined by WHO as a ‘delay in acceptance or refusal of vaccines despite the availability of vaccination services’ (Macdonald, 2015). In contrast, the opposite state is termed ‘vaccine confidence’. In dominant public health discourses, vaccine hesitancy has been seen simply to reflect lack of knowledge of vaccine benefits and (mis) perceptions of their risks amongst publics, due to their ignorance. Hesitancy in this conceptualisation thus reflects a ‘deficit’ in public understanding, to be filled by communication (Gregory and Miller, 2000). More recently this notion has shifted to embrace excess of information, seeing publics as confused and gullible to mis- and disinformation, conspiracy theories and the influence of an increasingly-globalised anti-vaxx movement. The challenge then shifts towards replacing ‘wrong’ with ‘right’ information and restoring perceived lack of trust in public health institutions. This shift from a deficit model to what one might term an ‘infodemia’ model in the public understanding of science is reflected in the WHO's recent focus on ‘infodemic management’ (WHO, 2021). In this vein, ‘demand creation and communication’ appears as the tenth area of WHO's vaccine readiness tool.

Longstanding social science literature brings a far more socially and politically embedded picture of vaccine engagements, as we discuss below. In brief, it shows that public concerns and controversies around vaccination are as old as the technology itself (Allen, 2007; Durbach, 2004), reflecting diverse, context-specific understandings and socio-political experiences (Larson, 2020). Yet despite social science research, negative, narrow discourses about vaccine hesitancy persist and indeed have gained new power around Covid vaccines: first, given the rapidity and novel technologies of Covid vaccine development it is assumed that publics will see them as more risky; and second, vaccines have been caught up in the so-called infodemic around Covid-19 in general, so that publics are seen as more exposed than ever to misinformation and conspiracy. The problems with these discourses on ‘hesitancy’ remain: whilst the anti-vaxx movement may prey on and amplify peoples' vaccine concerns, it cannot be considered simply to cause them - publics are neither blank nor fickle slates, ready to absorb and act on the last message they receive. Furthermore, whilst social media might now enable messages to travel in networks across the globe, these discourses do not fully recognise that particular concerns and ‘conspiracies’ are shaped by political environments and people's own interpretations of vaccine policies and politics – such as around their manufacturing origins and safety assessments.

In this regard, what is underplayed in both these sets of debates – about Covid vaccine supply and distribution, and demand and hesitancy – are located dynamics and experiences, in particular national and local settings in Africa, and the more nuanced, socially- and politically-embedded perspectives associated with these. Moreover, the separation of supply and demand-side issues in current debates overlooks possible interconnections between the two.

## 2 Conceptualising and exploring vaccine anxieties

In this paper, we ask: How are Covid vaccines understood and experienced in particular African social contexts? In what ways have vaccine supply and distribution affected demand or hesitancy? How are people's perspectives and experiences shaped by political dynamics across scales, from local to global? To address these questions, we draw on and expand the vaccine anxieties framework (Leach and Fairhead, 2007) developed around vaccine controversies in the early 2000s. This is part of a large, longstanding anthropological and historical literature on responses to medical technologies and health interventions in African contexts, to which this paper further contributes. Works focusing variously on clinical trial participation (eg. Geissler and Molyneux, 2011), blood sampling (eg. White, 2000; Fairhead and Leach, 2006), pharmaceuticals (Reynolds Whyte et al., 2002), interventions (eg. Beisel et al., 2016) and epidemics including Covid-19 (Charters and McKay, 2020), have explored the meanings, interpretations and practices of diverse publics, and the social, political and historical (including colonial) contexts in which these are embedded. The logics underpinning so-called ‘rumour’ and ‘resistance’, and the dynamic interplay with experience and pragmatism, have been key debates in this wider work to which our analysis contributes.

Our vaccine anxieties framework treats anxiety as polysemic. Used in a negative sense it implies a state of unease, worry or concern, yet it also has a positive meaning, implying an earnest, focused desire for something (Leach and Fairhead, 2007: 3). We thus re-cast vaccine hesitancy/confidence as vaccine anxieties, negative or positive, thereby allowing exploration of who, in which contexts, really does want Covid vaccines, and may be worried about not getting them. The anxieties framework emphasises people's agency, and does not assume that they passively accept vaccination or anti-vaxx messages. Instead of focusing on the deficits - what people do not think or understand - it emphasises what they do think and understand, as part of their everyday lives, values and conceptualisations of the issues involved, and how that influences action. Rather than judge understandings as right or wrong, it considers how they make sense in relation to people's contexts and experiences. Extending the 2007 formulation, the anxieties framework allows us to link supply and demand, exploring for instance how people's vaccine anxieties may be linked to experiences of navigating access to a scarce resource, or perceptions of which vaccines are being made available by and for whom. As we show, procurement and supply are far more complex, politicised and unpredictable than current debates about global vaccine justice often imply, and vaccine anxieties are deeply linked to how people interpret and navigate these uncertainties.

The vaccine anxieties framework as originally developed encompasses several interconnected dimensions, building on and connecting strands of anthropological and social science work on vaccination, health and science: Bodily dimensions – exploring how the body and aetiology of health and disease are understood and experienced and the place of vaccination in this (eg. Lock and Nguyen, 2018; Samuelson, 2001); social dimensions - exploring the social worlds that vaccination becomes part of, involving relations within families, communities, clinics, and health systems (eg. Nichter, 1995; Streefland et al., 1999), and wider political experiences – exploring people's encounters and imaginings about health and related institutions nationally and internationally, and the broader political and economic histories and governance regimes in which they are embedded (eg. Feldman-Savelsberg et al., 2000, Vaughan, 1992). In this paper we address the anxieties of local publics in African settings about Covid vaccines through a lens that attends to these bodily, social and wider political dimensions. A major aim is to bring to light people's own framings, showing how they make sense in their particular contexts. In so doing, we draw attention to significant gulfs that exist between such contextualised framings, and the over-simplified (mis) representations in dominant global discourses that reify supply/justice issues on the one hand and demand/hesitancy issues on the other. We consider how these might be bridged by focusing on dynamics at the intersection – bodily, social and political. An analysis of vaccine anxieties then leads us to a reworked, expanded notion of vaccine preparedness that goes beyond a technical approach to procurement, distribution and information management, to incorporate a more nuanced understanding that interlinks supply, delivery and demand, considers how these interlinkages could be anticipated, and attends to longer-term, contextually-embedded social and political relations.

## 3 Methods and sites

This study forms part of a broader collaborative anthropological research programme on pandemic preparedness in Africa, involving research in global and regional settings, the latter from the regional hub of Senegal, and in national and local sites in Uganda and Sierra Leone. These countries were chosen because they are both identified as ‘hotspots’ for disease threats and have relevant experience of Ebola, sharpening the interest of government and other agencies in preparedness. Their contrasting recent political experiences and regional positioning also offered valuable comparative contexts for the broader programme. Both countries share similar Covid-19 vaccine supply contexts, relying significantly on COVAX with some influence also from China, and both have experienced Ebola vaccine trials. The programme has focused predominantly on Covid-19 and from 2021, as vaccination started to be discussed and then implemented in the study countries, this became a focus of fieldwork and analysis.

The paper draws on documentary, social media and policy analysis at global and regional levels, on participant observation in regional meetings, and in Uganda and Sierra Leone, on ethnography involving informal, open-ended interviews in two rural villages and/or a peri-urban context in each country, complemented by observations in their respective provincial/regional urban centres. In Uganda, one village is in Kasese District, western Uganda and Gulu municipality constitutes a second site in northern Uganda. In Sierra Leone, one village is in Kailahun District, Eastern Province and a second in Moyamba District, Southern Province. Focused data collection on vaccine anxieties took place from January–September 2021, by research officers, the first of whom had been living and conducting participant observation in villages since August 2019. Field notes from all sites were shared weekly by email, with a combination of regular team discussion and written and oral feedback highlighting areas to explore, provided by colleagues with long term experience of doing ethnographic research in Uganda and Sierra Leone respectively. The locally-focused sections below draw directly on informants’ statements collected as part of this work. However with broader village fieldwork and interpretation still ongoing, and with field teams and supervisors able to connect only remotely, this paper does not claim to provide fully-analysed medical ethnographies.

We begin with a discussion of the global and regional situation regarding Covid vaccine supply in Africa as it unfolded during the first eight months of 2021. This sets the scene for country case studies of vaccine anxieties in Uganda and Sierra Leone. These explore the interactions between country-specific vaccine procurement and delivery, and the understandings and experiences of people in our fieldsites during the same period, organised according to bodily, social and wider political dimensions.

## 4 Covid vaccines in Africa: global-regional-national dynamics

### 4.1 Supply challenges: Africa at the sharp end of vaccine inequity

By the end of July 2021 the biggest vaccination campaign in history was well underway, with more than 4.16 billion doses administered across 180 countries (Randall et al., 2021). While at this point 49 doses of Covid-19 vaccine had been administered per 100 people in the world, the average vaccination rate in Africa was five doses per 100 individuals, with the situation still concerning by the end of September 2021 at 10.9 doses per 100 (Statista, 2021; Kyobutungi, 2021). The highly unequal global distribution is glaring, and further divides are evident between African countries.

Africa CDC moved early in 2020 to participate in platforms enabling clinical trials for large vaccine development initiatives, a move praised as an act of solidarity for which its director, John Nkengasong, was honoured by the Gates Foundation (Gates Foundation, 2020). Since vaccine roll-out became possible at the end of 2020, wealthy countries have dominated in securing the lion's share of vaccine stocks, with early deals struck between pharmaceutical companies and governments. For Africa, high hopes were pinned on collaborative initiatives, notably the WHO-led COVAX through which around 600 million vaccine doses were expected to reach the continent by the end of 2021, a figure already revised by early July (Winning and Obulutsa, 2021). What began as an ambitious project has fallen short on many counts (Usher, 2021), not least wealthy countries' slow, limited financial contributions and their own vaccine nationalism, limiting available vaccines for global distribution. In January, Nkengasong warned that growing inequity in access to Covid vaccines would mean that Africa would become the ‘Covid continent’, drawing a parallel to the slow access to antiretroviral drugs during the worst of the AIDS pandemic (Spinney, 2021). This reference to the past raised old charges of Africans as ‘guinea pigs’ in scientific trials but not equal beneficiaries of their products, a charge also raised in protests in francophone countries (Tilley, 2020) and underscored by resistance to the sharing of vaccine Intellectual Property. As the first quarter of 2021 brought new waves of Covid-19 on the continent, the urgency to access vaccines grew. However, supplies were disrupted in March when India – whose Serum Institute had been contracted by COVAX to manufacture and supply Astra Zeneca (AZ) technology vaccines (called Covishield) – pre-empted supplies to address its own national Covid crisis. Notwithstanding alarm about vaccine evasion by the Beta variant, globally the focus on variant surveillance and vaccine development as the key to managing Covid in the future intensified, as evidenced by the emphasis of WHO COVID-19 Roadmap meetings during this period.

In attempting to redress the slow arrival of vaccines, the supply picture that unfolded in 2021 was complicated by multi-polar geo-politics as China, Russia and other bilateral providers by-passed UN-led initiatives to supply African countries directly. By June, Chinese provision to Africa exceeded that from COVAX (Development Reimagined, 2021). A growing number of Africa-led initiatives also emerged, including the African Union's common platform for vaccine procurement, and the Africa Vaccine Acquisition Task Team (AVATT) project aiming to provide vaccines to 400 million Africans through World Bank-supported loans and engagement of a private bank (World Bank, 2021a). By June 2021, Africa-based vaccine production projects with donor support were emerging as part of a longer-term vision for greater continental self-reliance, for instance in Rwanda, South Africa and Senegal (European Investment Bank, 2021).

These multiple procurement routes have had consequences for vaccine supplies at country level; these have been unpredictable in their quantities and timing, and involved different vaccines with different technologies, origins, and financial and political implications. As we shall see, vaccine anxieties have often turned on how these uncertainties have been navigated nationally and locally, and how publics have perceived them.

### 4.2 Early vaccine readiness efforts

Such supply shortages were perhaps not anticipated when global vaccine readiness activities focused on Africa began, as early as August 2020. The WHO Africa office then created a Covid Vaccine Task Force, with the support of Africa CDC. To prepare for roll-out at scale, countries were requested to develop vaccination plans covering coordination, funding, legislation, logistics, communication and community engagement. However by January 2021, the readiness self-evaluation tool (VIRAT/VRAF) developed by this Task Force showed an average score of 35% for the continent, when 80% was expected. Many countries were found to lack trained health professionals, resources and monitoring procedures (Achieng, 2021). Mamta Murthi, the World Bank's Vice President for Human Development, put this delay down to Africa not being ‘used to vaccinating their adults’ (The Development Podcast, 2021). As revealed during participant observation in regional meetings, many countries delayed joining COVAX until at least December 2020. Some were cautious about the feasibility of maintaining the cold chain. Some feared the burden of implementation costs (estimated at five times the vaccine cost). Others were uncconvinced of the relevance of vaccines for their own populations, having experienced low Covid prevalence and mortality in 2020, reflecting the so-called ‘African paradox’, and assuming that this sparing would continue (Ghosh et al., 2020). Agencies such as UNICEF expressed concern that Covid vaccination campaigns risked diverting resources from longstanding vaccination efforts for other diseases, compounding the slowdown in routine vaccination already evident due to pandemic measures. Furthermore, WHO's Vaccine Readiness Assessment Tool used in all 47 countries in the WHO African Region showed that just 12% had plans to communicate with communities; WHO thus feared that populations would not be sufficiently informed to accept vaccines (WHO Africa, 2020).

### 4.3 Concerns about vaccine hesitancy

During late 2020, as part of vaccine ‘readiness’, several agencies conducted large scale surveys of vaccine perceptions amongst African populations. Five major surveys covered over 12 million respondents in 22 countries. These included an Africa CDC survey conducted during September–December 2020 in 15 countries, as part of its COVID-19 Vaccine Development and Access Strategy (Africa CDC, 2021b). This claimed a high overall acceptance rate, with 79% of respondents stating that they would agree to take a Covid-19 vaccine if it was deemed ‘safe and effective’. However, other surveys indicated lower acceptance, challenging this crucial framing qualifier. Bardosh et al. (2021) review the key themes that emerged at the time from these surveys, along with media and social media. Attitudes towards vaccination were affected by popular understandings of the virus: its origins, perceived risk profile, ideas of natural immunity and the impact of variants on vaccine effectiveness. Concerns were also raised about safety, side effects, rushed vaccine trials and new technologies. Speculation was evident about a lack of accessibility on the continent due to limited funds or corruption. Fears about possible forced vaccination also featured.

### 4.4 Dynamics at the interface of supply and demand

During the first eight months of 2021 our regional fieldwork and observations in meetings raised further issues that confounded neat plans. These are indirect consequences of vaccine shortages in Africa but illustrate the pivotal interconnections between questions of supply and demand. In turn, these issues become evident in vaccine anxieties, as we will go on to show for Uganda and Sierra Leone.

First, at the beginning of 2021, the vaccine shortage in African countries was generally managed, as in wealthy countries, by targeting priority populations (usually health workers, and people over 60 or with co-morbidities). However, for various reasons – including pressures from other groups, health authorities’ anticipation of higher vaccine supplies arriving, and lower-than-expected demand by priority populations - some countries were quick to open vaccination to the general population. In Senegal, for example, this happened after only four weeks, when priority groups were far from completely vaccinated (World Bank, 2021b). This shift had several consequences: deterring targeted outreach activities; deterring national communication strategies, lest they encouraged vaccine demand beyond available stocks; and opening the way for in-country disparities in vaccine distribution, as scarce stocks became focused on accessible/urban areas or elite groups. Uganda exemplifies a country that mandated vaccination as a condition of employment for groups such as teachers, causing further pressure to access limited supplies and anxiety, as we detail below.

Second, issues emerged around the expiry of vaccine doses and their relocation between countries. Malawi's destruction of just-expired doses in May 2021, when vaccination rates were low and Covid prevalence high, was justified by the government as reassuring the public that any doses administered were safe, and deterring perceptions that they might get inferior vaccines (Odhiambo, 2021). When news first broke that DRC and South Sudan had destroyed vaccine doses that they could not deliver, global agencies expressed major concerns that such ‘vaccine wastage’ would counter the call for more vaccines for Africa. The Vaccine Task Force set up a dose monitoring system and mechanism to oversee the recall and redistribution of doses nearing expiry towards countries in need. This created a new discursive category of countries at risk of dose expiry, presumed due to ineffective vaccine delivery and/or high levels of vaccine hesitancy. Eligibility for vaccine relocation involves legal and technical proof of ability to deliver, bringing interventions to shift potential host countries from ‘high risk’ to ‘high uptake’, as for Cote d’Ivoire in May 2021. However, the status of relocated doses is often kept opaque. Senegal, for instance, was supposed to receive doses from DRC, but did not admit this publicly, presumably to deter public anxieties about depreciated vaccines. Yet amidst prevailing vaccine nationalism, such unpublicized deals are easily leaked and interpreted through rumour as evidence of clandestine political alliances.

Third, the combination of multiple suppliers, disrupted global supplies and procurement-through-relocation created situations of high diversity of vaccines available at any time or in succession. This created chaotic circumstances in Senegal, for instance, as people navigated a sequence of AZ, Sinopharm and Janssen, trying to obtain a corresponding first and second dose and resorting to whatever was available as a third covid wave in July sparked a rush. Such dynamics occurred increasingly in various African countries, leading WHO to issue a warning on July 13 against ‘mixing and matching vaccines from different manufacturers’ and that ‘it will be a chaotic situation in countries if citizens start deciding when and who will be taking a second, a third and a fourth dose’ (Farge and Revill, 2021).

Fourth, globally emerging science and media coverage of side effects grew as vaccination programmes progressed apace in high-income countries. In March 2021, blood clots associated with AZ vaccines became a dominant topic in international news. The narrative that the AZ vaccine was suitable for low-income settings due to easy refrigeration and its related promotion for COVAX, combined with the safety issues flagged in the US and also in Europe where there were concerns about which age groups should receive it, reinforced the idea of it as an inferior vaccine (Dyer, 2021). This led to speculation that it was being ‘dumped’ on African populations, as was evident from our fieldwork in Uganda. This fieldwork also illustrates how shifts in regulatory bodies' concerns and later travel regulations that accepted certain vaccine brands and not others as valid protection, also shaped how different vaccines were perceived. For example, distinctions drawn by some European governments between Indian and European-manufactured AZ vaccine caused speculation about inferior vaccines coming through COVAX to African countries.

## 5 Covid-19 vaccine anxieties

In this context of vaccine shortages and complicated procurement issues, we now go on to explore vaccine anxieties as they unfolded during January–September 2021 in national and local settings, first in Uganda and then in Sierra Leone. In keeping with the vaccine anxieties framework we explore why certain publics did or did not want Covid vaccination, amidst their understandings of the disease; of social and community dynamics, and of wider political issues surrounding Covid vaccines. Extending the anxieties framework, we show how the supply and procurement dynamics discussed above, as they have played out nationally and locally, were interpreted by different people such as to become part of their vaccine anxieties, while also affecting their experiences of accessing vaccination. We also explore how anxieties changed over the period in relation to shifts in both disease and vaccination experiences.

## 6 Uganda

### 6.1 National context

Vaccination has long been central to Uganda's broad health policy framework and plans (Akello and Beisel, 2019), especially for childhood diseases. The Ministry of Health (MOH)'s Expanded Programme on Immunisation (EPI) started in the early 1970s and became a formal policy in 2014, targeting polio, tetanus, TB, pertussis, measles and rubella for the under-fives. In the mid-2000s, this minimum package was broadened to include pneumococcus, HPV, Hepatitis B and influenza, also broadening the age groups targeted for vaccination. For instance, individuals over 18 are now offered Hepatitis B vaccine if they report to state-aided health centres.

Uganda also has an emergency health threat framework which is mobilised to address epidemics. Typically a National Task Force is established, chaired by the prime minister, to call for and distribute emergency funds and measures. This was the approach used to prepare for the anticipated 2018 Ebola epidemic (Akello and Parker, 2021), and to respond to Covid-19. Covid measures have included a series of highly restrictive and militarised lockdowns and forcible hospitalisations, implemented in 2020 in a context of political oppression surrounding national elections, and in which symptomatic Covid cases and mortality in Uganda remained very low – thus damaging livelihoods and fuelling resentment and distrust amongst local populations (MacGregor et al., 2021; Parker et al., 2020). Covid-19 vaccinations have been rolled out against this backdrop, with the procurement and distribution of vaccines overseen by the National Task Force.

The moment Uganda received its first batch of COVAX-donated 864,000 AZ vaccines, in March 2021 (UNICEF, 2021), the President offered a national address in which he specified who would be eligible for the vaccine. This was ‘high risk’ populations, defined as the military, health workers, the elderly (defined as people over 50 years old) and people sick with other conditions such as HIV/AIDS. Notably, the president ordered the armed forces to get vaccinated against Covid-19 at the earliest opportunity. Children were not eligible. As we show below, in a context of limited experience of the disease and yet a heavy-handed state response, significant negative anxieties about Covid vaccination began to circulate.

The situation became more complex from March 2021, when Uganda diagnosed its first Covid-19 patient with the delta variant and experienced a virulent second wave. From March to July 2021 many patients experiencing this variant were severely ill and death rates increased in the capital city, Kampala, as well as Entebbe and Gulu. Fear of Covid-19 increased, fuelled by shortages of hospital beds, health personnel and therapeutic treatments including medical oxygen. Amidst this second wave, positive anxieties for Covid vaccines started to increase, especially in the most visibly-affected urban areas. At the same time, the government made vaccination mandatory for teachers and health care professionals, creating anxieties to become vaccinated to avoid losing one's job. Yet this higher vaccine appetite coincided with what were by then acute shortages of vaccines in the country. In Kampala and Wakiso districts which were most affected, health officers re-called vaccines distributed to villagers, so that they could offer them to vaccine-hungry urban populations. A group of unauthorised individuals sought to profit from the situation by mounting a fake Covid vaccination exercise, in which at least 800 vaccine-hungry people were persuaded into paying for ‘vaccines’ which were largely water (Rédaction Africanews, 2021) In contrast, people in our rural and peri-urban study sites remained hesitant to access even the (highly limited) AZ supplies available. There are bodily, social and wider political dimensions to these anxieties, which we now explore.

### 6.2 Bodily dimensions

Vaccination is a highly familiar medical technology in Uganda. Yet participants questioned the idea of vaccination for a disease that at least until the second wave, was of questionable existence – a ‘disease of the radio’ as some put it. Even once Covid-19 did become more visible as a disease in the second wave, supply shortages meant that the vast majority of people had not been vaccinated. In this context, some continued to express views that vaccines were not necessary. As one man put it ‘you can see that Covid has found me here alive and I will continue living. Why take the vaccine … … … ‘.

Bodily anxieties also turned on possible adverse effects from Covid vaccination. For some, this was linked to personal bodily experience, and particular reactions. Thus one young woman explained her hesitancy to access Covid vaccines because of recent life-threatening experiences with penicillin injections, stating ‘I have no interest of accepting the vaccine because it could make me die’. Another villager reported a relative's worries that vaccination would worsen her heart condition. Such personal stories and associated fears of the Covid vaccine were shared and amplified through mobile phone messaging and communication with friends and relatives.

More generally, some people worried about possible Covid vaccine side effects given their novelty. People living in peri-urban Gulu expressed concern that the vaccine was linked to severe Covid and increased the risk of death. They gave the example of a nurse who experienced severe fever immediately after a vaccination and was rushed to hospital in a critical condition. In our study village in Kasese district, people also expressed concerns that their bodies might be overburdened by multiple vaccines. For example, Covid-19 followed closely on a hepatitis vaccination campaign in the village – suggesting, in the local language Lukonzo, that *bama hekya emibiri ye bindu binene* (we are making our bodies carry too many things). Those taking medication for other conditions expressed similar worries: one woman said ‘the face mask is enough for me because … people like me are having HIV/AIDS, so the vaccine will be putting extra drugs in my blood’. Another said ‘every month, I go to Bwera hospital to receive my drugs of high blood pressure. You can surely sympathize with me if I refuse to accept the Covid vaccine because should I accept, it means I am over burdening my body’.

Such bodily anxieties emerged in contexts where supply shortages meant people had minimal direct experience of Covid vaccination, and thus minimal opportunity to gain direct experience of its safety. Media coverage accentuated these anxieties, especially when it captured discussions taking place in Europe and the USA about adverse effects (such as blood clotting) from the AZ vaccine (Oduor, 2021; Menezes, 2021). Indeed, when the USA banned the administration of AZ, fear and hesitancy increased. This is hardly surprising - AZ was the only vaccine available in Uganda at the time.

### 6.3 Community/health service dimensions

While Covid-19 vaccines – in as much as they were available - were supposed to be administered by health care professionals employed by the MOH, people were well aware that this was part of a national, presidentially-led Covid response. In contrast to the widely accepted children's vaccination programmes, the distribution of Covid vaccines was associated with a heavy-handed and already-resented state Covid response (Akello, 2020; Parker et al., 2020). That soldiers were the first prioritised group, and allegedly took the vaccine with 100% compliance, only increased its association with a distrusted militarised state. At our Gulu field site, members of the village health team (VHT) who attempted to support the mandatory vaccination programme were viewed, in Acholi, as “*Lu dup dano*”, betrayers, aligned more with state interests than with local residents. Indeed, their involvement with the vaccine efforts initially created fear and anxiety. To quote one person: ‘The people who do the house-to house [visits] cannot easily be avoided. It can be shameful to avoid them … … …. “. Another person said, ‘I can feel shy refusing to do what the VHT tells me to do when my eyes are looking into his eyes … [Yet] we know in our hearts that vaccinating us is putting us in a trap. I hear that those people who are vaccinated are the ones who are attracted to the disease and are dying … ….“.

Amidst uncertain supply, Covid vaccination thus provided a lens through which people reflected on and expressed anxieties about their positions and relations within the wider community, and with local health services. At our fieldsite in Kasese district, people discussed matters such as the categorisation of priority groups and the perceived disorganisation of the health services. Some speculated that a Covid vaccination certificate would in future be required for journeys and worried that these were not always available. A particular issue for health care workers concerned the challenges of receiving and then rapidly allocating vaccine doses close to expiry, and of finding sufficient people to use up a given batch once unsealed. Government targets and threats of sanctions related to district health system performance are likely to increase pressures. The policy of mandatory vaccination for designated groups instigated debate in both research sites, such as regarding the motivations of government authorities, as discussed below.

### 6.4 Wider political dimensions

Anxieties about the local administration of Covid vaccines interplay with people's wider experiences of the state and its interventions, both around Covid-19 and more broadly. Concerns already existed about the use of public health programmes for nefarious purposes, particularly among politically and economically marginalised populations (Parker et al., 2008). At study sites in northern and western Uganda, the government's response to Covid accentuated these concerns. Protracted periods of lockdown, arbitrary arrests and enforced hospitalisation of asymptomatic cases merely fuelled discussion about whether or not the government's underlying intention was to poison particular ethnic groups or anti-government individuals; and whether the Covid vaccines would impair fertility and enable the government to take the land of affected groups. One elderly person reflected ‘they are targeting old people so that they die. I think government is betraying us - the aged - so that we cannot live longer’.

There were additional concerns about the vaccines on offer. Chinese products are often perceived to be low quality in Uganda. When Sinovac began to be rolled out in August 2021, and teachers were asked to come forward for this vaccination, there was considerable disquiet. In Gulu municipality, for example, many teachers support opposition parties and people were concerned that they were being targeted for sinister reasons. The situation was not helped by the UK government's decision not to recognise fully vaccinated Ugandans as ‘vaccinated’ when entering the UK. Such policies served to validate local narratives and discourses suggesting that Covid-19 vaccines donated from mainly G-7 nations to Uganda are less effective (Africa CDC, 2021a).

By August 2021, views began to change. More and more people made their own empirical observations about the impact of Covid on the health and well-being of themselves and their families. Anti-vaccine rhetoric decreased and many anti-vaccine campaigners started to lose ground. A pastor in our Kasese study site who spent eight days in hospital described how he almost died of Covid, and would now devote his energies to educating villagers about the benefits of vaccination. Similarly, a male villager said: ‘I have already taken the vaccine. I used the first vaccine that was brought at the subcounty; this brother of mine also took the vaccine. We took the vaccine on the same day because we know that Covid is dangerous. Many of our people in this village will take the vaccine because they now know that Covid is there.’ Thus anxieties about the dangers of the disease appeared to be starting to co-exist with and in some cases to override negative anxieties about vaccination.

### 6.5 Sierra Leone

#### 6.5.1 National context

Sierra Leone has a long history both of vaccination and of global connection via the trans-Saharan and Atlantic trading systems. Variolation was documented as a local method for control of yaws and smallpox as early as the 18th century. The word in the national lingua franca (Krio) for vaccination is *maklet* (derived from the obsolete English word “maculate”, to mark the skin).

*Maklet* is associated mainly with vaccination in childhood against diseases such as measles. Many older villagers recalled having *maklet*, and mothers still take their children for such injections today, run as part of the EPI programme by the Ministry of Health and Sanitation through its extensive network of village public health units. Thus, the general principle of vaccination to prevent disease is well known and understood. Yet many explained that *maklet* was something exclusively associated with children. Injecting adults against an emergent, global viral disease was a new experience, and troubled many. Worries were easily stirred by warnings against vaccination sent on social media by family members living overseas.

Older people also reflected on earlier vaccination campaigns such as for smallpox. Whilst many recalled the sometimes rather painful way in which vaccines had been delivered, there was often an acceptance that if the government ordered it then vaccination was something to be endured. They also recalled noticing that mass killer diseases such as smallpox had disappeared, and that risks from diseases such as measles and polio had sharply declined, subsequent to the application of mass vaccination.

The national Ebola epidemic in 2014–15 left a rather different mark. There was (then) no vaccination against Ebola. An Ebola vaccine trial (EBOVAC) in Kambia District (2015–2022) was received in mixed ways: some volunteers were motivated to join for reasons included altruism, hope of finding employment and desire for medical benefit, whilst rumours about exploitative “white” technology served as negative factors (Tengbeh et al., 2018). Efforts in community engagement were made to improve relations between citizens and vaccine trial personnel (Dada et al., 2019) and an Ebola vaccination programme is now being rolled out to protect front-line health workers. However Ebola left a broader legacy on attitudes to public health via the harsh procedures applied to isolate suspected cases and quarantine communities, including excessive spraying with chlorine, from which it is locally understood that many patients died. The use of excessive use of force to control public health is now a source of anxiety, and objection.

It was in this context that Covid-19 vaccines began to be distributed from early 2021.

Sierra Leone initially received supplies of AZ under COVAX and Sinopharm via agreements with China, a large-scale investor in mining in the country. The vaccines were initially offered through hospitals and health centres in the main urban centres, on a first-come first-served basis. Sinopharm was offered to all over-18s, and AZ reserved for the over 40s with underlying health conditions, such as hypertension and diabetes. There was widespread government messaging over the radio about the two vaccines, their origins and their allocation to their respective user groups.

This first roll-out ended by June, when supplies were exhausted. The MoHS states it administered about 140,000 first shots - less than 5 percent of the adult population. The national target was 20 percent by the end of 2021. New supplies were then accessed, including about 300,000 doses of the Janssen ‘one shot’ vaccine, and a second roll-out of the Covid-19 vaccination programme was launched in the last week of August. This was planned to include rural areas. Many villagers had been unable to afford the time or money to travel to urban centres during the first roll-out, particularly as supplies were not assured. So for the second roll-out 600 vaccination teams were trained and equipped to deliver rural vaccinations in situ – including in our two study villages. Yet uptake has been relatively low. A reason widely given by officials for this low take up is the spread of false information about the dangers of the vaccine from family members living abroad. Yet the variation between locations equally open to international social media messaging, and villagers' own accounts, suggest a more complex and diverse set of vaccine anxieties, which can again be grouped according to bodily, health service and wider political dimensions.

#### 6.5.2 Bodily dimensions

Some villagers drew on their experience with *maklet* to express an understanding that vaccination strengthened the body and thus prepared it to fight Covid-19. Thus a woman in our first fieldsite explained: “The nurses told me that [this] *maklet* fights a lot of illnesses in the body. For instance, the polio vaccine they are giving prevents blindness and paralysis. So, we as mothers, are encouraged to come up with our children”. Others were worried by the idea of a Covid vaccination for adults, since, as a male villager put it, “*maklet* only comes for babies and not adults”.

However, two examples of urban dwellers vaccinated in the first roll-out show how experience could transform views, even to the extent of attributing to their injection almost miraculous powers. One woman was taken to hospital with a respiratory disease. Her Covid test was negative; the doctor nevertheless recommended that she be vaccinated. At first the woman refused but the doctor insisted, and she states that she placed herself in God's hands. Within hours her respiratory condition improved, perhaps due to the antibiotics she had been administered, and she left hospital convinced that the vaccination had cured her, a fact she widely proclaimed to customers at her market-place fish shop. Another woman explained that she had missed the vaccination team by being away when they arrived, and then fell seriously sick a few days later. She recognised that if she had taken the vaccination she and her family would have blamed her sickness on it. Meanwhile, her husband had been vaccinated, with no ill effects. This encouraged her to separate vaccination and a contingent infection, and she now looked forward to taking the vaccine when next offered.

Given such understandings of Covid vaccines as curative, it is not surprising that those who hesitated over vaccination often explained that they were not ill. This was often an argument offered by the young. One young village woman confidently proclaimed that she needed no vaccine because she was “healthy and good looking”.

Very few villagers in either site claimed to have ever seen a case of Covid-19. Low rural transmission, at least in the first wave, limited community testing, and easy confusion of mild Covid symptoms with other diseases, such as flu and the common cold, all contributed to this situation. If Covid-19 did exist, some villagers reflected, it was common and mild, and probably something they had lived with all their lives.

In contrast Ebola (which also mimicked other more common diseases in its early stages) was so devastating in its later stages that it revealed itself as something new and terrifying. One village woman told us “most people saw what [Ebola] did and there was no way to deny”. Ebola required extraordinary steps to address a frightening new disease. By contrast, Covid-19 seemed not to be a new and serious disease at all; a male villager commented that “all the illnesses [that were here] before have [now] been taken as signs and symptoms for Corona, so how can people trust an illness [like that]?” He was a vaccine refuser, and implied he felt no need to run the risk of taking an unproven vaccine to prevent or cure something that had always been around.

Given such understandings, it is not surprising that many people at first were influenced by messages circulating in social media and amongst relatives around the dangerous side effects from a novel vaccine. Many villagers were well aware of the origins of the different vaccines, including that AZ had been manufactured in India, but that later supplies were from the UK. The multiple vaccines available led to speculation about their differences. Thus, in our first fieldsite, one man said “we are getting information that the vaccine is in two types. The one type is easy and simple with no [side] effects and the other is not easy and gives lot of side effects”. A female villager had “heard [from other villagers] that the vaccine is in three types. The first type does not kill, the second type cures and the last type kills”.

Yet over time, villagers found no evidence of severe adverse consequences among those they knew had been vaccinated. Gradually, although limited by the slow roll-out and poor vaccine access in rural areas, negative anxieties about side effects began to diminish as direct bodily experience began to contradict international social media messaging.

#### 6.5.3 Community/health service dimensions

In phase one of the vaccine roll-out in Sierra Leone, many villagers were disturbed to think that perhaps another Ebola was upon them. Covid, however, was less readily apparent than Ebola, and villagers reacted negatively to lock-down control measures when seeing little impact of Covid-19 on their daily lives. Rural people often commented on the impossibility of reaching an urban vaccination site. But this was less an expression of feelings of rural neglect, than, at that stage, more a ready-made excuse for not accepting vaccination. Once the second phase of roll-out took more account of the problem of access, villagers had to decide for themselves whether they would accept the vaccinations now available to them.

In some places, there is evidence of mass absenteeism when the vaccination team arrives. In others there is an enthusiastic turnout. The variations seem to relate mainly to the existing state of trust in government medical services. In the August roll-out, vaccine teams included staff of the village medical centres, so vaccination was being administered by personnel well-known to villagers. Some older villagers had already explained the fears generated in earlier vaccination campaigns when a team of complete strangers turned up and ordered bodies to be presented for *maklet*. This was a lesson the MoHS had learnt well from past experience. Several nurses reported positively on this familiarity factor as a reason for their success in persuading large numbers of potentially hesitant villagers to take the vaccine.

Yet the situation appears to be different in villages where there have been tensions between health personnel and the local community. Both our study villages have experienced such disputes, leading to local boycotts of the health facility. This lack of confidence in the local health system was sometimes directly linked with negative vaccine anxieties: as one male elder put it “I doubt if they will send the vaccines to our village health post … do you think people like us in the village can take it, when it is all over social media that its kills?“.

Tellingly, it is also in such settings that stories about harsh treatment under Ebola tended to surface. For example, a women's leader in a village with around 500 adults near our first fieldsite, where only 14 people turned out for the local vaccination exercise in late August 2021, explained that:

They are hesitant because of the bitter experience in the days of Ebola. Members of the burial team used to spray chlorine. Which led to the death of many people. So, many are still afraid simply because of what they saw and heard about people who died during Ebola. Most are thinking that the vaccine is poisoned.

#### 6.5.4 Wider political dimensions

Villagers often expressed concern that Covid-19 was a “political” disease, being used to manipulate outcomes in power contests at national or international level. Concerns that Covid-19 had been bioengineered by the Americans (or the Chinese) to weaken Africa, and especially to control population levels by reducing fertility, readily translated into hostility towards Covid vaccines. As one elder put it: “I'm thinking that the white men want to kill or reduce the African population …. [no] vaccines are manufactured in Africa; [and] there is a tendency that they will poison the vaccine, or send vaccines that are expired”.

Some people were anxious at first about Sinopharm, influenced by social media messages that the Chinese planned to exterminate African populations. However, these messages made little sense to many people, because Chinese investment in Sierra Leone is widely seen as beneficial, when Western countries have turned their backs. Accordingly, concerns quickly receded, especially after initial supplies were quickly exhausted. Some people stated that they were happy to accept any new vaccines provided they had been approved by WHO and the African CDC – reflecting confidence in global or pan-African institutions, rather than western countries.

Negative geopolitical ideas, while not fully disappearing, were then increasingly displaced by evidence that Covid-19 vaccination was not widely harmful and that side effects were mainly minor. Villagers who had earlier expressed negative anxieties sometimes freely admitted this change of perspective, based on a better understanding of the purpose of vaccination. As a male elder interviewed first in July explained on re-interview in September: “Even myself, I was initially afraid but I have changed my mind to finally take it … If I refuse now that will negatively impact the community. Above all, I want to build up my immune system”.

## 7 Discussion

Whilst it is easy to dismiss comments about vaccines as ill-founded, misguided misinformation and conspiracy – part of an infodemic – it is also the case that they often reiterate longstanding concerns about vaccination in Africa and globally; and they have been given new life by the specific supply issues around Covid-19. The cases of Uganda and Sierra Leone reveal that people's anxieties about Covid-19 vaccines – both negative worries, but also positive desires for the vaccines – are embedded in often sophisticated understandings and reflections that make sense amidst their social and historical contexts and experiences. Whilst we have broadly distinguished here between bodily, community/health service and wider political dimensions of these, it is clear that these categories are merely heuristic and that in practice, they overlap, with each informing the other.

The vaccine anxieties framework has enabled us to reveal these embedded logics, in ways that give weight to people's own ideas and practices. In extending the framework, we have explored interactions between supply and demand issues and the way in which political dynamics across scales influence access to and understandings of vaccination. We have also looked at changes over time, and how vaccine anxieties have been shaped by waves of disease, shifting supplies and changing exposure to vaccine experiences. Emerging insights include the ways that uncertain and stop-start supplies, and multiple vaccine types, interplay with anxieties and often fuel worries. Crucially, limited supplies also limit people's opportunities to become familiar with the vaccines, and gain confidence in them. Where Covid vaccine anxieties have shifted from negative to positive, this seems to reflect both growing experience with the vaccines, and growing experience and fear of the disease in the second wave – although in Sierra Leone, comparative experience with Ebola continues to support a perception that Covid-19 is relatively mild. Vaccine confidence also grew when the vaccines were delivered by trusted local providers – a situation more apparent in Sierra Leone than in Uganda, where people's experiences of local health services remained deeply entangled with their experiences of a heavy-handed Covid response. Yet even in Sierra Leone, local differences between villages in health service experience affected uptake.

In Uganda, mandatory vaccination of some groups remains a persistent source of worry. Yet people want vaccination certificates, and are frustrated when these are not available In Sierra Leone, without mandatory vaccination, it is a frustrating organizational detail that the second vaccine campaign struggles to match vaccine supply to availability of cards. People who in early 2021 were adamantly claiming that they would never take a Covid-19 vaccine were, by July, agitated over how and when they would be united with their missing vaccine certificates.

The observation that people's growing practical experiences with Covid-19 vaccines has tended to erode negative anxieties chimes with anthropological works on other novel pharmaceutical or preventive medical technologies in African settings which suggest that, over time, pragmatic responses can come to dominate and innovations can become socially normalised (eg. Reynolds-Whyte et al., 2002; Geissler and Molyneux, 2011). Yet our cases also highlight that as technologies enter everyday experience, they do not become ‘just’ technical; they remain subject to social and political meanings, and thus vulnerable to shifting social and political dynamics which may re-activate negative anxieties once more. Shifting disease dynamics can also complicate a straightforward path to confidence, as has been evident with Covid-19 as new variants have emerged that partly evade vaccines, complicating interpretations of efficacy which can be limited to prevention of severe illness but not necessarily infection. In the cases we have explored, these dynamics of attraction and worry depend on people's experiences and interpretations not just of the (vaccine) technologies themselves, but, crucially, on how and by whom they are supplied and delivered. Respectful dialogue that appreciates such perspectives is likely to be more effective in building vaccine confidence than top-down communication of ‘correct’ information.

The socially and politically-shaped interactions between ‘supply’ and ‘demand’ also mean that these concepts, and related ones such as ‘roll-out’, ‘deficit’ or ‘excess’, are far more than merely technical. Steady supply and broad-based access to vaccines is important not just to enable vaccine uptake, but also the building of confidence. Yet as our cases show, accessibility as locally experienced also depends on people's assessment of vaccine origins, the identities and trustworthiness of providers, and the presence or otherwise of socio-politically relevant material infrastructures such as certificates. Inclusion or exclusion of geographical areas or populations, or interrupted availability, or mandatory vaccination mandates, are interpreted politically. This underlines the importance of embedding vaccine accessibility in approaches that build trustworthy health systems for the long-term. This in turn means addressing global supply issues, towards overcoming a situation in which shortages and negotiated deals with different suppliers amplify the bumpiness of roll-out and are easily interpreted by publics as politically-motivated, amplifying distrust.

As second and third waves of Covid-19 with more severe mortality manifested in several African countries in 2021, the political pressure on governments to be on top of vaccination increased – while speculation about corruption in bilateral deals continued. In countries like Senegal, a focusing of political attention on vaccine readiness and procurement deals, also provided a useful screen for seemingly inadequate preparedness for the logistical challenges of testing and healthcare, as prevalence of the disease and mortality rose. Here as in many countries, a lack of resources in health systems and longstanding experiences of a political-economy of structural neglect hampered both vaccine roll-outs and epidemic preparedness and response in parallel. In this regard, diverting political attention in-country to supply and questions of global injustice can be useful to deflect attention from inadequate in-country capacities and inequities. Thus a focus on vaccine readiness as the central, technical key in epidemic preparedness and response can also be politically expedient - in de-emphasising those elements of preparedness that should be underpinned by stronger material, social and political infrastructures.

## 8 Conclusion

In this light, we need a new conceptualisation of ‘vaccine preparedness’ to become a central part of global health and emergency policy and planning. This must move beyond existing WHO notions of ‘vaccine readiness’ to address the longer-term structural, social and political relations in which vaccine delivery and distribution are embedded; and beyond narrow assumptions about vaccine demand or hesitancy to address the real anxieties both positive and negative felt by populations, embedded in bodily, social and wider political experience. It must encompass capacities for understanding and communication by health authorities that are less top-down and based on quantitative survey and data snapshots, and more socially-nuanced and interactive, based on respectful dialogue with community members, and attuned to the ongoing interplay of supply, distribution and anxieties, and the social and political meanings they carry, in rapidly-changing local, national and global contexts. And it will require vaccine policy and governance arrangements in country settings that are more inclusive and localised, able to foster trust and respond sensitively to anxieties, both longstanding and emerging. This will be important for continued efforts to address Covid-19, but also into the future as technological advances make vaccination an increasingly central part of epidemic preparedness and response more generally. Meanwhile, the embeddedness of vaccine anxieties in broader health system and governance contexts underlines that vaccine preparedness should not lead to neglect of other important parts of epidemic preparedness, including its structural underpinnings in strong, inclusive health systems.

## Credit author statement

Melissa Leach: Conceptualisation, Methodology, Writing – original draft preparation. Hayley MacGregor: Conceptualisation, Methodology, Writing – original draft preparation. Grace Akello: Supervision, Writing – original draft preparation. Lawrence Babawo: Supervision, Writing- Reviewing and Editing. Moses Baluku: Data collection. Alice Desclaux: Conceptualisation, Writing – original draft preparation. Catherine Grant: Data curation, Writing- Reviewing and Editing. Foday Kamara: Data collection. Marion Nyakoi: Data collection. Melissa Parker: Methodology, Supervision, Writing- Reviewing and Editing. Paul Richards: Supervision, Writing – original draft preparation. Esther Mokuwa: Supervision, Writing- Reviewing and Editing.. Bob Okello: Data collection, Writing- Reviewing and Editing. Kelley Sams: Writing- Reviewing and Editing. Khoudia Sow: Data collection, Writing- Reviewing and Editing.
